# A decade of violence and empty stadiums in Egypt: when does emotion from the terraces affect behaviour on the pitch?

**DOI:** 10.1007/s00181-023-02383-0

**Published:** 2023-02-15

**Authors:** Carl Singleton, J. James Reade, Dominik Schreyer

**Affiliations:** 1 Department of Economics, University of Reading, Whiteknights Campus, Reading, RG6 6EL UK; 2 Center for Sports and Management (CSM), WHU – Otto Beisheim School of Management, Erkrather Str. 224a, 40233 Düsseldorf, Germany

**Keywords:** Attendance, COVID-19, Football, Home advantage, Natural experiments, Referee Bias, Social pressure, C90, D91, L83, Z2

## Abstract

In less than a decade, the Egyptian Premier League has experienced three distinct changes between periods of competition in either crowded or empty stadiums. We exploit this unique sequence of natural experiments, to answer two questions neglected by the still emerging literature on the effects of crowds on behaviour and decision making. First, does reinstating a supportive crowd after a long period of absence affect performances on the pitch? Second, is any reduced home advantage from competing in empty stadiums robust to repeating such an experiment? We find that eliminating crowds decreased or even reversed home advantage after an incident of extreme crowd violence in 2012, but there were no significant effects when crowds were reinstated in 2018 and once more excluded in 2020.

## Introduction

On 1 February 2012, at the conclusion of an Egyptian Premier League (EPL) fixture between Al Masry Sporting Club of Port Said and Cairo’s Al Ahly Sporting Club (SC), a massive riot erupted et al. Masry Club Stadium, formerly known as Port Said Stadium, in which seventy-four people were killed and many hundreds more were injured.^1^ The event sparked a period of civil unrest over the following weeks. As a result, the Egyptian Government cancelled the remainder of the EPL’s 2011–12 season. The EPL returned for the 2012–13 season with empty stadiums, only for that season also to be cancelled due to a coup d'état (Reuters 2013). The EPL restarted for a full season in 2013–14 behind closed doors, i.e. in empty stadiums without any crowds. Spectators were to be allowed back into stadiums in early 2015 (Reuters 2014), before further violence led to another suspension and the continuation of the stadium ban (Reuters 2015). Eventually, the ban was lifted for the beginning of the 2018–19 season, though with restricted numbers, typically about 5000 spectators, and those attending had to provide their personal details to security services. In March 2020, the EPL was suspended because of COVID-19, like most football leagues were (see Tovar, 2021),^2^ before ultimately resuming, once again with empty stadiums for the remainder of the 2019–20 season and the entirety of the 2020–21 season. In this way, between 2012 and 2020, the EPL experienced three distinct changes between periods of either having crowds in attendance or playing in empty stadiums—twice when fans were shut out and once when at least some of them could return.

We exploit this series of natural experiments, occurring over a decade, to shed some first light on the question of what happens when spectators, who typically favour the team playing at home, not only leave the stands but also return after a relatively long period of absence. While the COVID-19 pandemic has induced a global natural experiment motivating economists and psychologists alike to study the causal effects of an absent crowd on performances in professional sports, including by the officials (e.g. Bryson et al. 2021; Endrich and Gesche 2020; Ferraresi and Gucciardi 2021; Fischer and Haucap 2021; McCarrick et al. 2021; Scoppa 2021; see also Reade et al. 2022, for an initial summary), two natural follow-up questions have so far been largely neglected. First, does reinstating a supportive crowd after a long period of absence also affect performances? Second, is any reduced home advantage from competing in empty stadiums robust to repeating the experiment?

Answering these two questions can help us to better understand how social forces affect individual economic behaviour (e.g. Akerlof and Kranton 2000; Becker and Murphy 2000; Bursztyn and Jensen, 2017), which is relatively easy to assess in the regulated and controlled context of sports competition (Bar-Eli et al., 2021; Chan et al. 2021; Singleton et al. 2021a, b). There is already an extensive and continuously growing economic literature on the potential effects of the social environment in sports on decision making, not least on the relationship between football crowds and the behaviour of referees and judges (see the survey by Dohmen and Sauermann 2016). The underlying dynamics though, alternating between periods with and without such crowds, and potentially inducing or removing social pressure, are hardly understood. There is also an open question about the cultural and context-specific consistency of social effects on behaviour, especially about the effects caused by a large crowd of spectators who are invested in a particular outcome in sport or at other types of events. For instance, from the already more than thirty peer-reviewed studies constituting the still-emerging literature on how COVID-19 affected home advantage in professional association football, to the best of our knowledge not one has included observations from matches played in Africa,^3^ despite the evidence so far suggesting that the observed effects might be highly context-sensitive (e.g. Benz and Lopez 2021; Bryson et al. 2021; Fischer and Haucap 2021).

## Background and data

Although the Egypt’s men’s national football team, also known as the Pharaohs, has only qualified for the FIFA World Cup three times (i.e. in 1934, 1990, and 2018), the country has a rich football pedigree and history. At the men’s senior international level, Egypt is the most successful football nation in the African continent’s history, having won the Africa Nations Cup seven of the thirty-three times it was held between 1957 and 2021, more than any other nation. Egypt’s dominance is even greater at the club level, where an Egyptian team has won the top continental club competition sixteen times since the first edition in 1964, including two of the last three editions in 2020 and 2021, and where the next most successful nation, Morocco, is currently on seven successes. Egypt has also had a notable recent impact on the elite European professional football leagues. For example, one of the best players in the world, Mohamed Salah currently of Liverpool F.C., is Egyptian and began his career with Al Mokawloon Al Arab SC in the EPL.^4^

However, when it comes to “Egyptian football”, perhaps the second thing that the average football fan thinks about, after Mohamed Salah, is violence. Despite filling large stadiums with tens of thousands of emotive fans in the past,^5^ more EPL games have taken place in empty stadiums than with fans in attendance in the last decade. Table 1 briefly summarises the recent history of this competition since the beginning of the 2009–10 season. As described in the introduction here, the Egyptian ultras, and all other football supporters, were first banned from the stadiums in February 2012 after the Port Said Stadium Riot, followed by an extension of that ban in 2015 because of more violence and a reinstatement in 2020 because of COVID-19.

**Table 1:** Egyptian Premier League (EPL), seasons 2009–10 to 2020–21

Period	Season	Teams	Number of EPL matches			Remarks
			Played	With crowds	Without crowds	
A	2009–10	16	240	240	0	
A	2010–11	16	240	240	0	EPL was suspended in January 2011 for over 3 months due to the Egyptian Revolution, but then restarted and completed by mid-July 2011
A	2011–12	16	143	143	0	EPL was suspended following the 1/2/2012 Port Said disaster and then not completed
B	2012–13	18	136	0	136	EPL split into two and two teams added. Was suspended in July 2013 due to Egyptian coup d'état and not completed
B	2013–14	22	227	0	227	EPL remained split in two and four more teams were added, with relegation and championship playoff games
B	2014–15	20	379	0	379	Fans were supposed to be allowed back into stadiums in early 2015, after the end of the Africa Cup of Nations (8/2/2015) but excluding matches involving 6 teams. However, there was a deadly incident at a match in Cario when fans attempted to force access to the stadium on 8/2/2015—thereafter, the season was completed with empty stadiums
B	2015–16	18	306	0	306	EPL returned to a single division and no play-off games
B	2016–17	18	305	0	305	Zamalek did not show up for a match at Misr Lel Makkasa
B	2017–18	18	306	0	306	
C	2018–19	18	306	306	0	Up to 5000 fans allowed back into stadiums, after providing personal details to security services
C/D	2019–20	18	305	156	149	Zamalek refused to play away at Al Ahly. EPL was suspended in March 2020 due to COVID-19 and later completed in empty stadiums
D	2020–21	18	306	0	306	EPL season was completely played in front of empty stadiums

**Fig. 1:**
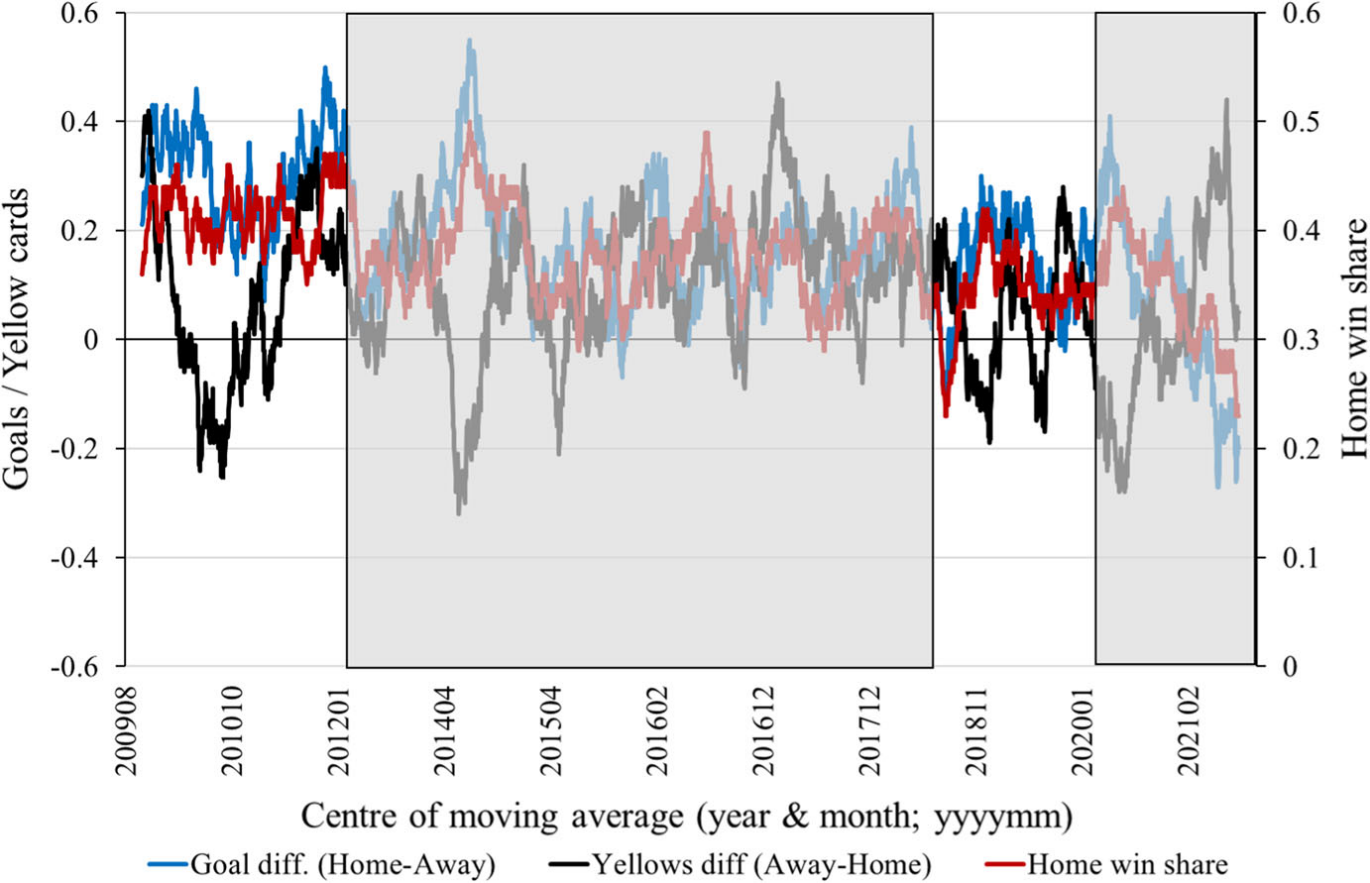
Average outcomes in football matches of the Egyptian Premier League, August 2009 to August 2021. Notes: author calculations using data from https://worldfootballdata.net, accessed 10/11/2021. Series show a centred moving average over 100 football matches of outcomes: share of matches ending in a home win; the average final match goal difference between the teams (home minus away); and the average difference between away and home in the total numbers of yellow cards awarded by referees within matches. Shaded areas denote the periods where matches were played in empty stadiums—see main text and Table 1 for descriptions of these periods. See Appendix Figs. A1–A3 for monthly averages instead of the centred 100-match moving averages

Information collected and verified using https://worldfootballdata.net, accessed 10/11/2021, and various other sources, including Wikipedia and news articles. ‘Period’ refers to differences over time in whether matches took place in empty stadiums (see also Fig. 1)

We use this period of variation in the presence or not of a stadium crowd to test whether it affects outcomes and behaviour on the pitch, either consistently or differently depending on the context. We collected data on the outcomes of all EPL football matches between the beginning of the 2009–10 season and the end of the 2020–21 season.^6^ Like much of the literature that has used the experience of COVID-19 and professional football without crowds to infer their impacts (e.g. Bryson et al. 2021), we focus on a few high-level outcomes of football matches that normally show an advantage to the team playing at home: (1) the match outcome and the difference this implies between teams in league points earned (home win $=>3$ points diff., draw $=>0$ points diff., away win $=>-3$ points diff.); (2) the goal difference between teams according to the final match score line; (3) the total numbers of yellow cards awarded to each team; and (4) the difference in these numbers of cards between the teams, calculated as away minus home such that a positive value suggests home advantage.^7^ The last of these outcomes captures the different extent to which the teams involved were punished for foul play and is often used in the literature to point towards referee bias (e.g. Dohmen and Sauermann 2016). The outcomes based on final match score lines are normally used to measure overall home advantage in football. Table 2 summarises the descriptive statistics for these match outcomes as well as stadium capacities in the EPL.

**Table 2:** Sample descriptive statistics for matches in the Egyptian Premier League, August 2009 to August 2021

	Mean/share	S.E.	Min	Median	Max
Home win	0.373	0.009			
Draw	0.330	0.008			
Away win	0.297	0.008			
Points diff. (Home-Away)	0.229	0.043	− 3	0	3
Goal diff. (Home-Away)	0.171	0.026	− 6	0	6
Yellow cards diff. (Away-Home)	0.081	0.029	− 6	0	6
Home yellow cards	1.746	0.022	0	2	7
Away yellow cards	1.827	0.023	0	2	7
Stadium capacity (000 s)*	30.918	21.537	1	25	86
*N* of all matches	3195				
*N* of empty stadium matches	2112				
*N* of open stadium matches	1083				

Author calculations using data from https://worldfootballdata.net, accessed 10/11/2021. All matches played in the sample period, with the earliest match being played on 6/8/2009 and the latest being played on 28/8/2021

*We were only able to collect the stadium location and capacity data for 2874 of the matches

Figure 1 illustrates the development of home advantage in the EPL from the beginning of the 2009–10 season to the end of the 2020–21 season, with the shaded areas covering the periods of empty stadiums. Largely reflecting a tendency observed in European football (e.g. Peeters and van Ours 2021), as well as in Cricket (Reade 2019) and the Olympics (e.g. Singleton et al. 2021a, b), among other sports and competitions, we note a slight decline in home advantage over time. In the first two years in our sample period, about 41% of matches were won by the team playing at home, compared with 35 per cent of matches in the last two years. Despite the series in Fig. 1 displaying centred moving averages of outcomes over one hundred EPL matches, there is stark and substantial variability over time in the differences between home and away teams in the numbers of goals and yellow cards awarded (see also Appendix Figures A1–A3 for month-by-month averages of match outcomes over the sample period).

## Estimation

To estimate the effects of playing football in an empty stadium, we assume that the conditional mean of the outcome $y_{ijm}$, in match *m,* involving home team *i* and away team *j*, is given by:

1 $$ E\left[ {y_{ijm} | C_{ijm} ,h_{i} ,a_{j} } \right] = F\left( {C_{ijm} ,h_{i} ,a_{j} ; \beta } \right), $$

where $C_{ijm}$ is a dummy variable taking the value of one when a match was played in an empty stadium and zero otherwise. Here, $h_{i}$ and $a_{j}$ are fixed effects for the home and away teams, respectively, consistent with an assumption that in some period there are general tendencies for some football teams to be relatively stronger than others when playing at home or away (e.g. Peeters and van Ours 2021; Pollard 1986, 2006). These fixed effects also address the unbalanced sets of matches played with and without fans in attendance in the estimation samples (Bryson et al. 2021). The parameter $\beta$, to be estimated, measures the effect of playing in an empty stadium on the football match outcome. In the ideal experiment to identify $\beta$ and interpret it as a causal effect, we would repeatedly hold the same football matches, involving the same players, officials, and other participants, and in the same conditions, except for varying the presence or not of fans in the stadiums. Although this is impractical, the fact that the stadium bans, and their later rescindments, were not driven by football reasons, could still allow for a causal interpretation of our estimates of $\beta$. This is the case so long as the home and away-team fixed effects can plausibly address the non-random variation in match outcomes, and in particular home advantage, driven by differences in the types of matches played with and without fans. As we discuss later, this is partly why we prefer to estimate Eq. (1) over relatively short periods of matches, as any football fan knows that the relative home and away strengths of teams tend to vary a fair amount over longer periods of time.

Nonetheless, we considered including on the right-hand side of Eq. (1) some other well-known determinants of the variation in football match home advantage that the fixed effects would not necessarily soak up, such as the distance travelled by the away team to a match (Clements et al. 2021; specifically for association football see Oberhofer et al. 2010),^8^ the precise scheduling of the match (e.g. Goller and Krumer 2020), and the recent form of the teams playing (e.g. Zhao and Zhang 2017). However, we found that variables addressing the former two of these potential determinants were generally statistically insignificant when included in the estimation of Eq. (1). We prefer to omit time-varying variables capturing the recent form of teams as there is no possibility that these could determine whether a match was played in an empty stadium. We would also be concerned that form variables, e.g. the dynamic Elo ratings or recent win percentages of teams, could become contaminated by the effects of playing in an empty stadium, especially close to the times in the EPL when the ability of fans to attend matches changed. We also considered including linear or quadratic time trends over days in all the models that we estimate. However, we found these to be statistically insignificant, conditional on the other explanatory variables, not only over the whole sample period but also within the shorter periods that we focus on later.

We estimate Eq. (1) using linear least squares when $y_{ijm}$ is either the points difference achieved by the teams in a match, the goal difference, the number of home yellow cards, the number of away yellow cards, and the difference between the away and home teams in the numbers of yellow cards issued to them by the referee. In particular, we estimate the following:

2 $$ y_{ijm} = \gamma + \beta C_{ijm} + h_{i} + a_{j} + \varepsilon_{ijm} , $$

where $\gamma$ is a constant and $\varepsilon_{ijm}$ is the error term. We also estimate Eq. (1) using Poison regression when $y_{ijm}$ is the positive count variable for either the number of yellow cards awarded to the home or away team. In general, and as in previous studies (e.g. Reade et al. 2022; Benz and Lopez 2021), we prefer the poison model for counts of yellow cards awarded to individual teams.^9^ We estimate Eicker–Huber–White heteroskedasticity robust standard errors for all models.

We select four different estimation periods: (I) whole sample period: August 2009 to August 2021; (II) before/after the Port Said Stadium riot, 2011–12 and 2012–13 seasons; (III) 2018, letting fans back in, 2017–18 and 2018–19 seasons; (IV) 2020, shutting fans out due to COVID-19, 2019–20 season only. Period (I) covers all three distinct changes in the presence or not of fans in the stadiums (see Fig. 1 and Appendix Figures A1–A3). Periods (II)–(IV) each cover one distinct change in the presence or not of fans in the stadiums. The choices of estimation periods were guided by the scheduling of EPL seasons and having enough matches to generate some statistical power, with the trade-off in the latter regard being the strength of the fixed home and away team effects assumptions then implied by the regression models.

## Results

Table 3 shows the estimated effects from Eq. (1) of playing in empty stadiums in the EPL instead of with fans in attendance, $\widehat{\beta }$, varying the estimation period and the dependent variable. Specifically, the columns give estimates of the effects for the four different sample periods of matches described above, and the rows give the effects for each of the five different outcome variables that we focus on. In column (I), for the whole period, the estimates point to reduced home advantage in terms of the points gap and goal difference between teams when fans were absent from the stadiums, though not statistically different from no effect at the 5% level. Over the whole period between 2009 and 2021, playing in an empty stadium was also associated with significantly fewer yellow cards being awarded to both the home and away teams, by 5–6%, or around one card fewer awarded to each team every ten matches.

**Table 3:** Estimated effects of playing football in empty stadiums in the Egyptian Premier League, August 2009 to August 2021

Dependent variable	Whole period (I)	2012: Before/after Port Said Stadium riot (II)	2018: Letting fans back in (III)	2020: Shutting fans out due to COVID-19 (IV)
*Least squares (*$\widehat{\beta }$*)*				
1. Points diff	− 0.102	− 0.521	0.280	0.271
(Home-away)	(0.092)	(0.268)	(0.221)	(0.253)
2. Goal diff	− 0.093	**− 0.408**	0.029	0.100
(Home-away)	(0.054)	(0.163)	(0.127)	(0.150)
3. Yellows diff	0.008	− 0.225	0.284	− 0.110
(Away-home)	(0.066)	(0.181)	(0.155)	(0.190)
4. Home yellows	**− 0.104**	0.087	**− 0.516**	-0.062
	(0.051)	(0.147)	(0.116)	(0.143)
5. Away yellows	− 0.095	− 0.138	− 0.232	− 0.172
	(0.052)	(0.145)	(0.125)	(0.144)
*Poisson: exp(*$\widehat{\beta }$*)-1*				
6. Home yellows	**− 0.057**	0.054	**− 0.273**	− 0.029
	(0.027)	(0.083)	(0.050)	(0.073)
7. Away yellows	− 0.050	− 0.070	-0.123	− 0.093
	(0.026)	(0.067)	(0.060)	(0.070)
*Estimation sample sizes*				
*N* of all matches	3195	279	612	175
*N* of empty stadiums	2112	136	308	149
*N* of open stadiums	1083	143	304	156

Author calculations using data from https://worldfootballdata.net, accessed 10/11/2021. See main text, Tables 1, 2, and 3, & Fig. 1 for descriptions of the data and estimation periods covered by the column headings. Rows 1–5 show least squares estimates of Eq. 1 for the effect of playing in empty stadiums. Rows 6 and 7 show Poisson model equivalent estimates of Eq. 1 for the numbers of yellow cards, where the estimates displayed should be interpreted as the % reduction in cards awarded to either the home or away team when matches took place in empty stadiums. All models have home-team and away-team fixed effects. Robust standard errors for the estimates are shown in parentheses. Bold indicates estimates that are significantly different from zero at least at the 5% level, using two-sided tests

In Appendix Table A1, instead of the empty stadium dummy variable in Eq. (2), we show estimates of models over the whole period with dummy variables for the four different sample periods, as highlighted in Table 1 and Fig. 1. In the base Period A, before the Port Said stadium riot and with fans, there was significant home advantage in the points gap and goal difference, but not in yellow cards. For the latter outcome, there was no significant difference in the later 3 periods compared to Period A. For the whole post-Port Said riot period (B) with no fans attending, there were significant reductions in home advantage in terms of the points gap and goal difference compared with Period A, by 0.26 points and 0.15 goals. Also relative to Period A, there were even greater and statistically significant reductions in home advantage both in Period C (letting fans back in) and Period D (shutting fans out due to COVID-19). While looking at these patterns over the whole sample period is somewhat insightful, it is potentially confounded by other factors that changed distant from the switches in whether fans were attending, such as rule changes, the setup of the League, and varying relative team home and away strengths.

Column (II) of Table 3 shows the estimates of Eq. (1) considering only the EPL seasons either side of the Port Said Stadium riot in 2012. The estimated reduction in home advantage associated with this event and playing football in an empty stadium was substantial, with a large but statistically insignificant reduction in the points gap by 0.5 and a significant reduction in the goal difference by 0.4, which effectively turned the normal home advantage into an away advantage. For comparison, when European football clubs had to lock out stadium crowds due to COVID-19 restrictions during the season 2019–20, Scoppa (2021), for instance, found that home/away teams obtained only about 0.1 points per game less/more when playing in empty stadiums. Other authors, in contrast, found no such robust significant effect on match outcomes (e.g. Bryson et al. 2021), or, if so, only in some leagues (e.g. Benz and Lopez 2021; Fischer and Haucap 2021). Rather, a reduction in home advantage was more likely to show in a decrease in (yellow) cards awarded to the away team (e.g. Endrich and Gesche 2020), a finding that seems to generally hold if teams must play in empty stadiums only temporarily (e.g. Pettersson-Lidbom and Priks 2010). Despite this, there appears to have been no significant effect of the empty stadiums in this period on the numbers of yellow cards awarded to either the home or away teams.

However, it is worth noting that our statistical tests in these sub-sample analyses are lacking in power. The insignificant reduction in the points gap by 0.5 from an empty stadium in the first row of column (II) (*p*-value = 0.053) is at least equivalent to the magnitude of the pre-riot home advantage in the EPL (see Appendix Figure A1). The same applies to the insignificant estimated reduction in the yellow cards difference of 0.2 in the third row of column (II) (*p*-value = 0.216) (see Appendix Figure A3). In general, our power analysis for the various models suggests that our estimates of *β* in the sub-sample analyses would need to be at least as large as the magnitude of home advantage prior to the Port Said Stadium riot for us to find them statistically significant, in terms of the points gap and goal difference, and substantially larger than that magnitude for yellow cards.

Columns (III) and (IV) of Table 3, respectively, focus on the two periods around letting fans back into stadiums for the start of the 2018–19 season and shutting them out because of COVID-19 within the 2019/20 season. Around both these events, the estimates of Eq. (1) suggest no evidence of a significant change in home advantage without fans for points earned and goals scored. Compared with the previous season, the 2018–19 season saw significantly more yellow cards awarded to home teams when fans returned to the stadiums (column (III), row 4 & 6), which also points towards a positive effect of empty stadiums on home advantage in these later periods.

Over the period that we study, several matches in EPL were played in neutral stadiums or even neutral cities, even though they still had a designated home and away team. It is also a concern for the interpretation of the results in Table 3 that the EPL has a few teams with substantially larger supporter bases than others, to the extent that some away fixtures could have had greater numbers of supporters of the away team in attendance than for the home team (e.g. Al Ahly SC playing away against other teams who are also based in Cairo), especially since we understand that stadiums were far from filled to capacity before the bans. We would expect these characteristics of EPL to bias the estimates of Eq. (1) towards no effects on home advantage from playing in an empty stadium.

We could find no reliable data on attendances by home and away team supporters for the EPL. Therefore, as a robustness check in Table 4, we use only matches where the home team was playing in their home city and the away team travelled outside the city where they were based, to estimate all the same variants of Eq. (1) as summarised in Table 3. In theory, this sub-sample of matches ought to have featured on average a greater preference towards the home team among the fans attending the stadiums, thus partially addressing the aforementioned bias to the estimated effects of playing in an empty stadium. The results in Table 4 confirm this, especially for the period around the Port Said Stadium riot. After dropping the matches where home teams were not playing in their home cities and away teams were not travelling between cities, the estimated reductions in home advantage from playing in an empty stadium are greater (column (II), rows 1–3).

**Table 4:** Robustness checks—only matches where home team played in home city and away team travelled to the city

Dependent variable	Whole period (I)	2012: before/after Port Said Stadium riot (II)	2018: letting fans back in (III)	2020: Shutting fans out due to COVID-19 (IV)
*Least squares (*$\widehat{\beta }$*)*				
1. Points diff	− 0.205	**− 0.885**	0.417	0.274
(Home-away)	(0.128)	(0.410)	(0.359)	(0.367)
2. Goal diff	**− 0.148**	**− 0.507**	0.060	0.185
(Home-away)	(0.075)	(0.250)	(0.209)	(0.214)
3. Yellows diff	− 0.070	− 0.549	0.333	− 0.135
(Away-home)	(0.092)	(0.299)	(0.255)	(0.280)
4. Home yellows	− 0.001	0.338	**− 0.351**	0.093
	(0.071)	(0.251)	(0.173)	(0.217)
5. Away yellows	− 0.070	− 0.211	− 0.019	− 0.042
	(0.071)	(0.223)	(0.184)	(0.215)
*Poisson: exp(*$\widehat{\beta }$*)-1*				
6. Home yellows	0.000	0.212	**− 0.206**	0.055
	(0.040)	(0.141)	(0.081)	(0.113)
7. Away yellows	− 0.038	− 0.118	− 0.009	− 0.029
	(0.036)	(0.095)	(0.098)	(0.112)
*Estimation sample sizes*				
*N* of all matches	1716	156	317	171
*N* of empty stadiums	1053	60	163	80
*N* of open stadiums	663	96	154	91

See Table 3. All models have home-team and away-team fixed effects. Robust standard errors for the estimates are shown in parentheses. Bold indicates estimates that are significantly different from zero at least at the 5% level, using two-sided tests

We also check whether the empty stadiums effects are heterogeneous according to stadium capacities. A larger capacity would suggest higher attendances before the stadium bans, potentially greater social pressure from the home crowd, and thus the possibility of a larger loss in home advantage in an empty stadium. However, most of the stadiums in the EPL, especially the larger ones, have a running track, which increases the distance between the fans and the pitch. This fact has the potential to reduce home advantage (e.g. Buraimo et al. 2010), and thus, the effects of playing in an empty stadium as capacity increases. In Appendix Tables A2 and A3, we show results equivalent to Table 4 (i.e. still dropping the matches where home teams were not playing in their home cities and away teams were not travelling between cities), whereby we split the estimation samples according to whether matches were played in stadiums with above or below the median capacity of 25,000 over the whole sample period. Although even more under-powered than in our earlier results, this analysis suggests that the reductions in home advantage predominantly occurred in lower capacity (smaller) empty stadiums in Egyptian football. Likewise, the results suggest that the reinstatement of a reduced group of fans may only have a positive effect on home advantage in small stadiums. Potentially, this could relate to the dispersion of the crowd within a stadium affecting the extent of social pressure that it exerts, or the effects of a small crowd likely being further from the pitch in a small stadium.

Unfortunately, we do not have information on the balance of home and away fans in the Egyptian stadiums, to determine whether size or composition of the crowd matters most for home advantage. For example, evidence from Colella et al. (2018) shows that removing only the away part of the crowd in Argentinian football tended to increase home advantage. Humphreys et al. (2022) also find some tentative evidence that the away attendance in English football could particularly affect football match outcomes.

## Discussion and conclusions

Adding to the still-emerging literature on the causal effects of stadium crowds on human behaviour and decision making, our findings indicate that banning such crowds from the stands seems to reduce home advantage (c.f., Reade et al. 2022), at least initially. When playing in empty stadiums in the aftermath of the 2012 Port Said Stadium riot, EPL’s teams experienced a significant reduction in both the previously positive points gap and the goal difference when playing at home. This finding is generally consistent with or even somewhat greater in magnitude than what recent studies have found from exploring European association football played in empty stadiums (behind closed doors), either occasionally (e.g. Pettersson-Lidbom and Priks 2010) or regularly (e.g. Bryson et al. 2021). However, there was no significant increase in the points gap or goal difference in favour of the home teams once the (limited) crowds were let back in at the beginning of EPL’s 2018–19 season. Similarly, we observe no further significance reduction in home advantage once spectators were banned due to COVID-19 in 2020. However, and as noted above, our statistical analysis of these latter two events is somewhat under-powered. Taken together though, our results suggest that, ultimately, not all home crowds seem to benefit the home team,^10^ perhaps because the degree of social pressure from once very emotional crowds might cool down while under observation and scrutiny.^11^ An alternative and related explanation might point to changes in the composition of EPL crowds over the studied period. As Rommel (2021) observes, by 2018 and before the return of (restricted) crowds in EPL’s stadiums, both Egyptian masculine fan groups and ultras movements had largely dissolved, which may have resulted in a substantial reduction in social pressure on the performances of the referee and other officials from the less vocal remaining home-team supporters in the crowds.^12^

Despite their potentially low external validity,^13^ our findings also add to the discussion of whether home advantage in football and other professional sports was eroded during COVID-19. While most previous authors have documented an often-substantial decline in home advantage after March 2020 across numerous European football competitions, the reported effect sizes tend to vary significantly across studies. This may not only reflect different estimation techniques but even more so potential sample selection biases (e.g. Benz and Lopez 2021). As Fischer and Haucap (2021) have noted, among others, one such confounding factor might relate to heterogeneity of crowd sizes before COVID-19. They found that the home advantage of German clubs from lower tiers, and thus with relatively small crowds, was larg^2022^ely unaffected by the COVID-19 induced spectator ban, whereas the impact on home advantage in the top tier appeared to be substantial. Our findings echo these previous ones (e.g. Reade et al. ), as well as pointing to another explanation for some of the observed heterogeneity in the effects of empty stadiums—the potential for substantial variance in crowd composition, not only across leagues but also over time within leagues. As such, we believe that exploring the effects of such crowd compositions (e.g. away/home crowds, violent radicalisation) rather than crowds, per se, would be valuable.

## Figures and Tables

**Fig. 2:**
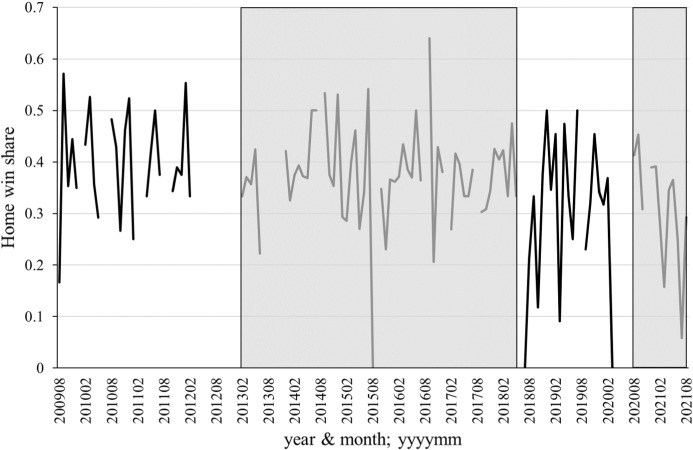
Monthly home win share for football matches in the Egyptian Premier League, August 2009 to August 2021. *Notes*: author calculations using data from https://worldfootballdata.net, accessed 10/11/2021. Shaded areas denote the periods where matches were played in empty stadiums—see main text and Table 1 for descriptions of these periods

**Fig. 3:**
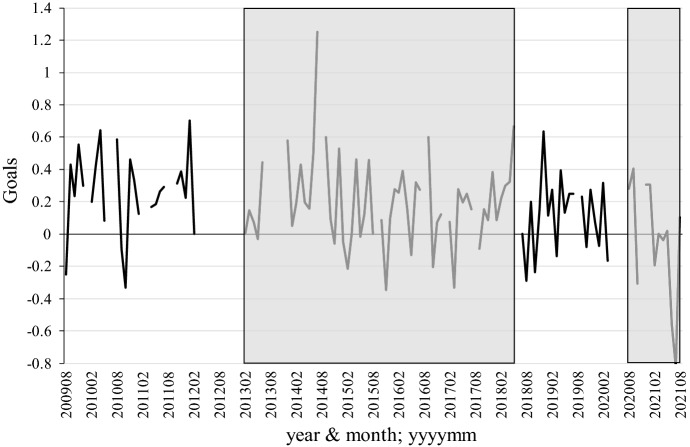
Monthly average goal difference (home-away) for football matches in the Egyptian Premier League, August 2009 to August 2021. *Notes*: author calculations using data from https://worldfootballdata.net, accessed 10/11/2021. Shaded areas denote the periods where matches were played in empty stadiums—see main text and Table 1 for descriptions of these periods

**Fig. 4:**
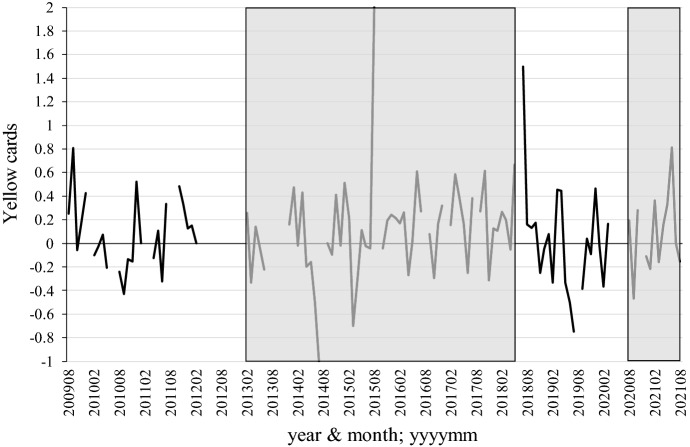
Monthly yellow cards difference (away-home) for football matches of the Egyptian Premier League, August 2009 to August 2021. *Notes*: author calculations using data from https://worldfootballdata.net, accessed 10/11/2021. Shaded areas denote the periods where matches were played in empty stadiums—see main text and Table 1 for descriptions of these periods

## Appendix A: additional tables & figures

See Tables 5, 6, and 7 and Figs. 2, 3, and 4.

**Table 5:** Period effects models for different match outcomes in the Egyptian Premier League, August 2009 to August 2021

	Points diff. (Home-Away) (I)	Goals diff. (Home-Away) (II)	Yellows diff. (Away-Home) (III)	Home Yellows (IV)	Away Yellows (V)
Constant	**0.476**	**0.306**	0.067	**1.821**	**1.889**
(Excl. cat. is Period A)	(0.098)	(0.058)	(0.070)	(0.056)	(0.055)
Period B	**− 0.259**	**− 0.145**	0.033	-0.119	− 0.086
(After Port Said riot)	(0.116)	(0.068)	(0.083)	(0.066)	(0.150)
Period C	**-0.408**	− 0.164	0.029	-0.020	0.009
(Letting fans back in)	(0.150)	(0.088)	(0.110)	(0.084)	(0.190)
Period D	**− 0.378**	**− 0.250**	− 0.048	− 0.081	− 0.129
(Shutting out – COVID-19)	(0.160)	(0.094)	(0.112)	(0.091)	(0.144)
*R*^*2*^	0.184	0.211	0.038	0.039	0.041
*N* of all matches	3195	3195	3195	3195	3195
*N* of empty stadiums	2112	2112	2112	2112	2112
*N* of open stadiums	1083	1083	1083	1083	1083

Author calculations using data from https://worldfootballdata.net, accessed 10/11/2021. See main text, Tables 1–3, & Fig. 1 for descriptions of the data and estimation periods covered by the column headings and variable names. Each column shows least squares estimates of Eq. (2), for the whole sample period, where the empty stadium dummy variable (${C}_{ijm}$) is replaced by period dummies according the four regions in Fig. 1 and as described in Table 1. All models have home-team and away-team fixed effects. Robust standard errors for the estimates are shown in parentheses. Bold indicates estimates that are significantly different from zero at least at the 5% level, using two-sided tests

**Table 6:** Robustness checks—only matches where the home team played in their home city, the away team travelled to that city, and the stadium was relatively small (< 25,000 capacity)

Dependent variable	Whole period (I)	2012: Before/after Port Said Stadium riot (II)	2018: Letting fans back in (III)	2020: Shutting fans out due to COVID-19 (IV)
*Least squares (*$\widehat{\beta }$*)*				
1. Points diff	− 0.315	**− 2.221**	0.884	− 0.238
(Home–away)	(0.185)	(0.795)	(0.660)	(0.577)
2. Goal diff	**− 0.220**	− 0.800	0.611	0.030
(Home–away)	(0.106)	(0.483)	(0.424)	(0.320)
3. Yellows diff	− 0.212	− 0.702	0.473	− 0.198
(Away–home)	(0.127)	(0.577)	(0.538)	(0.442)
4. Home yellows	0.138	0.316	− 0.634	0.045
	(0.099)	(0.370)	(0.328)	(0.339)
5. Away yellows	− 0.074	− 0.387	− 0.161	− 0.156
	(0.102)	(0.460)	(0.411)	(0.337)
*Poisson: exp(*$\widehat{\beta }$*)-1*				
6. Home yellows	0.082	0.201	**− 0.353**	0.018
	(0.059)	(0.210)	(0.119)	(0.143)
7. Away yellows	− 0.041	− 0.176	− 0.092	− 0.104
	(0.052)	(0.151)	(0.193)	(0.170)
*Estimation sample sizes*				
*N* of all matches	854	80	118	82
*N* of empty stadiums	511	16	58	31
*N* of open stadiums	343	64	60	51

See Tables 3 and 4. 25,000 is the median stadium capacity for the whole sample period (see Table 2). All models have home-team and away-team fixed effects. Robust standard errors for the estimates are shown in parentheses. Bold indicates estimates that are significantly different from zero at least at the 5% level, using two-sided tests

**Table 7:** Robustness checks—only matches where the home team played in their home city, the away team travelled to that city, and the stadium was relatively large (≥ 25,000 capacity)

	Dependent variable	Whole period (I)	2012: Before/after Port Said Stadium riot (II)	2018: Letting fans back in (III)	2020: Shutting fans out due to COVID-19 (IV)
*Least squares (*$\widehat{\beta }$*)*	1. Points diff	− 0.049	− 0.263	− 0.146	1.005
	(Home-away)	(0.187)	(0.663)	(0.263)	(0.504)
	2. Goal diff	− 0.063	− 0.374	0.245	0.388
	(Home-away)	(0.111)	(0.453)	(0.301)	(0.319)
	3. Yellows diff	0.089	− 0.508	0.473	− 0.065
	(Away-home)	(0.141)	(0.608)	(0.538)	(0.463)
	4. Home yellows	− 0.125	0.240	− 0.266	0.106
		(0.111)	(0.505)	(0.212)	(0.337)
	5. Away yellows	− 0.036	− 0.268	− 0.021	0.041
		(0.103)	(0.335)	(0.211)	(0.322)
*Poisson: exp(*$\widehat{\beta }$*)-1*	6. Home yellows	− 0.067	0.149	**− 0.138**	0.093
		(0.057)	(0.212)	(0.102)	(0.173)
	7. Away yellows	− 0.018	− 0.134	− 0.006	0.028
		(0.053)	(0.135)	(0.108)	(0.156)
*Estimation sample sizes*					
*N* of all matches		859	69	198	83
*N* of empty stadiums		540	41	105	46
*N* of open stadiums		319	28	93	37

See Table 3 and 4. 25,000 is the median stadium capacity for the whole sample period (see Table 2) All models have home-team and away-team fixed effects. Robust standard errors for the estimates are shown in parentheses. Bold indicates estimates that are significantly different from zero at least at the 5% level, using two-sided tests

## Footnotes

1 For a more detailed summary of the events that night, we refer to Wikipedia (bit.ly/portsaidriotw). For video footage of the riots, including players being attacked by fans on the pitch (bit.ly/portsaidriots), and how the event sparked violence on the same night in other football stadiums (bit.ly/cairostadium), we refer to the YouTube channel of the national British daily broadsheet newspaper The Telegraph.

2 In Europe, a minority of football leagues, including the Danish Superliga, the Swiss Super League, and the Russian Premier League, eventually returned to complete their campaigns with strictly restricted stadium attendances (e.g., Cueva 2020). See Reade et al. (2021) for a study of an exceptional professional football league in Belarus that continued with fans in stadiums throughout the early stages of the Covid-19 pandemic.

3 The tendency to ignore African observations in this context is by no means surprising, given that most previous research in the field of sports economics has focused on exploring either European or US settings (e.g., Gomez-Gonzalez et al. 2021; Schreyer and Ansari 2021).

4 At the time of submission, November 2021, European football pundits and professionals were commonly referring to Mo Salah as the “best player in the world” (e.g., Daily Mail 2021; Eurosport 2021; Guardian 2021).

5 Unfortunately, we were unable to find reliable match-level data or even season-level averages for Egyptian stadium attendances before the Port Said disaster in 2012. However, Egypt currently has 18 football stadiums with at least a capacity of 20,000. The modern Borg el Arab stadium in Cairo holds 86,000, and before it became an all-seater, the Cairo International Stadium could hold 120,000 standing, doing so for the 1986 Africa Nations Cup final.

6 We collected these data from https://worldfootballdata.net.

7 Throughout, the analysis, we group yellow cards awarded to players for their first offence in a match with those awarded for a second offence that would also result in a red card.

8 Typically, the literature suggests that fatigue, particularly following considerably long air travel and jet lag, tends to increase home advantage. As a counterexample, though not from football, Zak (2021) finds that Chess players perform significantly better when they travel abroad for tournaments, pointing toward the greater incentives to perform after investing in costly travel.

9 We prefer Poisson (quasi-maximum likelihood estimator; QMLE) to the negative binomial regression because the former is efficient in the class of consistent estimators with under or overdispersion (variance/mean ratio is constant) for effects on the conditional mean, provided it is correctly specified (Silva and Tenreyo, 2006; Wooldridge 2010). In other words, the Poisson QMLE estimator does not require the dependent variable to have a Poisson distribution. It is also robust to different forms of heteroskedasticity and measurement error, as well as being well behaved for large fractions of zeros in the estimation sample (Silva and Tenreyo, 2011). We also considered a Skellam distribution for the differences between two dependent Poisson or general count random variables (i.e., goals or yellow cards) (see Karlis and Ntzoufras 2003), but this also appears to offer no overriding benefits to estimate the effects of interest on the conditional mean outcome.

10 Somewhat related, Colella (2021) observes a significant increase in the performances of non-white players, relative to white players, when Italian supporters were banned from stadiums due to COVID-19 in the 2019–20 season.

11 Note that, as indicated in Table 1, those (few) fans that were allowed to enter the stadiums in 2019 had to provide personal details to security services.

12 Related, Goumas (2014) concluded that for European club continental football the density of a crowd affected referee decisions, and not the size of the crowd nor its proximity to the pitch once density was controlled for.

13 As one reviewer has rightly argued, the Egyptian football environment appears more violent than the typical sports league in most Western countries.
